# Predicting involuntary hospitalization in psychiatry: A machine learning investigation

**DOI:** 10.1192/j.eurpsy.2021.2220

**Published:** 2021-07-08

**Authors:** Benedetta Silva, Mehdi Gholam, Philippe Golay, Charles Bonsack, Stéphane Morandi

**Affiliations:** 1 Community Psychiatry Service, Department of Psychiatry, Lausanne University Hospital and University of Lausanne, Lausanne, Switzerland; 2 Department of Health and Social Action (DSAS), Cantonal Medical Office, General Directorate for Health of Canton of Vaud, Lausanne, Switzerland; 3 Epidemiology and Psychopathology Research Unit, Department of Psychiatry, Lausanne University Hospital and University of Lausanne, Lausanne, Switzerland; 4 Ecole Polytechnique Fédérale de Lausanne EPFL, School of Basic Sciences, Institute of Mathematics, Lausanne, Switzerland; 5 General Psychiatry Service, Department of Psychiatry, Lausanne University Hospital and University of Lausanne, Lausanne, Switzerland

**Keywords:** Coercion, involuntary hospitalization, machine learning, predicting factor

## Abstract

**Background:**

Coercion in psychiatry is a controversial issue. Identifying its predictors and their interaction using traditional statistical methods is difficult, given the large number of variables involved. The purpose of this study was to use machine-learning (ML) models to identify socio-demographic, clinical and procedural characteristics that predict the use of compulsory admission on a large sample of psychiatric patients.

**Methods:**

We retrospectively analyzed the routinely collected data of all psychiatric admissions that occurred between 2013 and 2017 in the canton of Vaud, Switzerland (*N* = 25,584). The main predictors of involuntary hospitalization were identified using two ML algorithms: Classification and Regression Tree (CART) and Random Forests (RFs). Their predictive power was compared with that obtained through traditional logistic regression. Sensitivity analyses were also performed and missing data were imputed through multiple imputation using chain equations.

**Results:**

The three models achieved similar predictive balanced accuracy, ranging between 68 and 72%. CART showed the lowest predictive power (68%) but the most parsimonious model, allowing to estimate the probability of being involuntarily admitted with only three checks: aggressive behaviors, who referred the patient to hospital and primary diagnosis. The results of CART and RFs on the imputed data were almost identical to those obtained on the original data, confirming the robustness of our models.

**Conclusions:**

Identifying predictors of coercion is essential to efficiently target the development of professional training, preventive strategies and alternative interventions. ML methodologies could offer new effective tools to achieve this goal, providing accurate but simple models that could be used in clinical practice.

## Introduction

Coercion in psychiatry is a widely discussed and controversial issue. In fact, although coercive measures are justified by the need to preserve the patient’s health and safety and to protect others from their potentially harmful behaviors [1], their use is in strong opposition to the principle of patient’s autonomy and self-determination also defended by the UN Convention on the Rights of Persons with Disabilities [2]. Furthermore, despite the increasing political and ethical attention to patients’ human rights and the scarce evidence of coercion’s benefits [3], its use is increasing almost everywhere [4–6] with great variations among countries [7,8]. A recent study compared the annual incidence of involuntary hospitalization for 22 countries across Europe, Australia, and New Zealand between 2008 and 2017 showing that time trends in annual rates raised in 11 out of 18 countries [6]. Moreover, Rains et al. [6] found that incidences varied strikingly, ranging from 282 per 100,000 inhabitants in Austria to 14.5 per 100,000 inhabitants in Italy. Higher annual incidence rates were associated with lower absolute poverty, higher gross domestic product and per capita healthcare expenditure, larger proportion of foreign-born individuals and higher number of psychiatric beds [6].

In previous studies, other area- and system-related characteristics have been associated with an increased risk of involuntary hospitalization. Rates of compulsory admission were higher in socioeconomically deprived and densely populated urban areas with higher proportions of young adults and ethnic minorities [9–12]. In addition, several aspects of the referral process, such as being referred outside regular service hours [13,14], by a general hospital [13,15], a general practitioner [15,16], or someone who does not know the patient [17], and having a contact with the police at the time of admission [17], were identified as predictors of involuntary hospitalization.

However, the most robust results point to the patient’s clinical profile as key determinant. People affected by schizophrenic, bipolar, or organic mental disorders, with poor insight, low treatment adherence, high level of psychotic symptoms and aggressiveness, and a history of previous involuntary treatment were more likely to be detained [3,11,14–35]. On the contrary, affective disorders, anxiety disorders, and substance-related disorders were associated with a lower risk of coercion [13,15,19,27,29,30,32]. Recently, a meta-analysis confirmed that previous involuntary hospitalization and diagnosis of psychotic disorders were associated with the greatest risk of involuntary psychiatric admission [36].

Among socio-demographic characteristics, being a young man, homeless, unemployed, with a migration background, and a low educational level were identified as being associated with involuntary admission [3,9,11,13,14,18,19,22,26,28,30,31,33]. However, these findings are less consistent, with several studies showing the opposite [16,25,37] or finding no association [15,20,27,32,38].

Most of the above mentioned studies aimed at identifying predictors of involuntary hospitalization using traditional statistical methods, mainly regression models [11,15–18,20,21,24–27,29–31,38], which made the statistical interaction between risk factors difficult to model and interpret [13,39,40]. Machine learning (ML) may overcome this limitation. ML algorithms are “trained” to find patterns in large amounts of data in order to make predictions based on new data and reveal previously “unseen” nonlinear interactions between variables [41,42]. A recent German study used a decision-tree-generating algorithm (CHAID Chi-Square Automatic Interaction Detector), to detect risk factors for involuntary admission on a weighted sample of 10,171 inpatients [13]. Beside main diagnosis, this study identified several modifiable predictors of involuntary hospitalization, such as the absence of outpatient treatment prior to admission or admission outside of regular service hours. Consequently, several preventive measures were suggested to address these factors and reduce the rate of involuntary inpatient treatment [13]. In 2020, Karash et al. [43] improved this first predictive decision tree model by optimizing ML techniques and broadening the dataset to include environmental socioeconomic data. Three main subgroups of patients were identified as at highest risk of involuntary admission: (a) patients with an organic mental disorder, retired, admitted outside of regular service hours, and living in assisted housing; (b) patients with suicidal tendencies but no affective disorder, with unclear previous suicide attempts, or living in areas with high unemployment rates; and (c) psychotic patients, living in densely built areas with a large proportion of small or one-person households [43]. ML algorithms were also employed by Günther et al. [41] to predict the use of coercive measures (seclusion, restraint, and involuntary medication) in a sample of 370 forensic offender patients with schizophrenia. Overall, 10 factors out of a set of 569 potential variables were identified as best predictors of coercion with a balanced accuracy of 73.3%. Previously, another Swiss study on 393 hospitalized patients had also shown that ML was useful in predicting coercion, reaching results comparable to binary logistic regression but with better generalizability [44]. Further studies were recommended by the authors to evaluate the potential of these methods on larger samples.

The purpose of this study was to use ML models to identify which characteristics predicted the use of compulsory admission on a large sample of psychiatric patients (*N* = 25,584).

## Methods

### Study setting

This study was set in the canton of Vaud (794,384 inhabitants in 2017), a mixed urban–rural region of Western Switzerland. The canton is divided into four psychiatric districts, each one served by a psychiatric hospital. In 2015, the four psychiatric hospitals provided a total of 0.6 psychiatric beds per 1,000 inhabitants [15], below the total number for Switzerland (0.9/1,000 inhabitants) and for Europe (0.7/1,000 inhabitants) [45,46]. On the contrary, according to data from the Swiss Health Observatory, in 2016 the canton of Vaud registered the highest rate of involuntary admissions in Switzerland (2.3 per 1,000 inhabitants), well above the national average (1.6 per 1,000 inhabitants) [47,48] and among the highest in Europe [6].

Involuntary admissions in Switzerland are regulated at federal level by the Article 426 of the Swiss Civil Code (CC) which states that *“a person suffering from a mental disorder or mental disability or serious neglect (the patient) may be committed to an appropriate institution if the required treatment or care cannot be provided otherwise.”*

Therefore, need for treatment is the legal requirement for involuntary admission. Lack of capacity to consent to treatment or the fact of posing an imminent danger to oneself or others are not required to decide on an involuntary hospitalization. However, danger to oneself or others is the prerequisite to detain a person suffering from a mental disorder who has voluntarily entered an institution and wishes to leave (Art. 427 CC). In this particular case, detention may last a maximum of 3 days, at the end of which the patient may leave the institution, unless a compulsory admission is ordered.

The federal law is then executed at cantonal level, with important regional differences. In the canton of Vaud, only the Adult Protection Authority (APA) and medical doctors designated by the Department of Health and Social Action, namely general practitioners, psychiatrists, pediatricians, and on-call doctors, are allowed to order an involuntary admission [49]. APA orders have no time limit, but a mandatory revaluation after 6 months, 12 months, and then every year. Instead, involuntary admissions ordered by medical doctors cannot exceed 6 weeks, unless the APA issues an extension order.

### Study design

This study retrospectively analyzed the routinely collected data of all psychiatric admissions that occurred between January 1, 2013 and December 31, 2017 in the four psychiatric hospitals of the canton of Vaud, Switzerland. Available data included socio-demographic characteristics, like gender, age, marital status, and nationality. Several clinical characteristics were also available, as primary diagnosis, aggregated into 10 main categories based on the ICD-10 system, a secondary diagnosis of addiction and/or personality disorders, and the 12 item-level scores of the Health of the Nations Outcome Scale (HoNOS) [50,51], which measure the patients’ mental and social functioning. Within the four hospitals, an additional observer-rated item on psychotropic medication compliance is also routinely assessed. This was also included in the analyses. Finally, we took into account the available information on the referral process, such as who referred the patient, to which hospital, the legal status of the hospitalization (voluntary or involuntary) and whether they were admitted during regular service hours (Monday/Friday from 8:00 a.m. to 18:00 p.m.) or outside regular service hours (Monday/Friday from 18:00 p.m. to 8:00 a.m., weekends and public holidays).

All data were anonymized and the Human Research Ethics Committee of the Canton Vaud (protocol #2016–00768) granted approval for this study.

### Statistical analysis

The included sample (*N* = 25,584) was split into two subgroups based on legal status of the hospitalization (voluntary hospitalization [VH; *n* = 15,797] vs. involuntary hospitalization [IH; *n* = 9,787]) and compared on all the available characteristics. Categorical variables were tested using Pearson’s Chi-square tests. Ordinal and continuous variables were analyzed through nonparametric Mann–Whitney *U* tests. All statistical tests were two-tailed. Because of the large sample size, significance level was set at *p* < .01 and effect sizes according to Cohen [52] were calculated.

In order to identify the main predictors of involuntary hospitalization, we used two ML algorithms, Classification and Regression Tree (CART) [53] and Random Forests (RFs) [54], on observations with no missing data (*N* = 14,948; 63.3% VH vs. 36.7% IH). Moreover, we used ordinary logistic regression to compare its predictive power with that obtained through CART and RFs.

First developed by Breiman et al. [53], CART is a flexible tool used to discover hierarchical and complex associations among variables. It is a nonparametric methodology, thus no assumptions are made on the distribution of the predicting variables and it can handle any type of data, numerical as well as categorical. Moreover, compared to traditional statistical methods, CART presents several advantages: (1) the best “splitting” variables are identified automatically at each step of the tree by searching among all available features; (b) it can easily deal with missing data; (c) it is relatively easy to perform, requiring little input from the user; and (d) its results are generally simple for clinicians to interpret [40]. CART presents a major improvement compared to previous tree-based algorithms, such as Automatic Interaction Detection (AID) and Chi-square Automatic Interaction Detector (CHAID). In CART, the tree can grow until further split is not possible due to the stop criteria, which can be defined a priori. For reasons of parsimony and generalization, we can also remove irrelevant branches by “*pruning*” in order to find the best potential CART [55].

RFs are a generalization of CART, in which a large number of trees are fitted on resampled versions of the original data and each single CART contributes to the final prediction by choosing the most important predictor.

Our predicted outcome was voluntary/involuntary hospitalization (VH vs. IH). IH was defined as the positive class and VH as the negative one. Socio-demographic, clinical, and procedural characteristics were the predictive variables (23 features; Table 1).

**Table 1.** Socio-demographic, clinical, and referral process characteristics: subgroups comparisons (*N* = 25,584).

Characteristics	VH (*n* = 15,797; 61.7%)		IH (*n* = 9787, 38.3%)		*p*-value	Effect size
**Age**, Mean (SD) Mdn (IQR)	42.3 (16.4)	41.0 (22.0)	48.6 (21.4)	46.0 (34.0)	<0.001^a^	*r* = −0.12^Δ^
**Sex**, Male, % (*n*)	48.5 (7,648)		49.3 (4,829)		0.217^b^	–
**Marital status, % (*n*)**						
Single	50.7 (7,947)		48.4 (4,694)		<0.001^b^	*V* = 0.11^Δ^
Married/registered partnership	21.5 (3,367)		21.0 (2,036)			
Divorced/separated	24.4 (3,822)		21.7 (2,102)			
Widowed	3.5 (553)		8.9 (865)			
**Nationality,** Swiss, % (*n*)	66.7 (10,479)		66.0 (6,425)		0.293^b^	–
**Primary diagnosis at discharge (ICD-10), % (*n*)**						
Organic, including symptomatic, mental disorders (F00–F09)	2.7 (429)		14.7 (1,433)		<0.001^b^	*V* = 0.22^ΔΔ^
Mental and behavioral disorders due to use of alcohol (F10)	10.4 (1,632)		9.3 (908)		0.003^b^	*V* = 0.02^Δ^
Mental and behavioral disorders due to psychoactive substance use (F11–F19)	9.4 (1,469)		4.8 (468)		<0.001^b^	*V* = 0.08^Δ^
Schizophrenia, schizotypal, and delusional disorders (F20–F29)	19.4 (3,041)		30.8 (3,007)		<0.001^b^	*V* = 0.13^Δ^
Manic and bipolar affective disorders (F30–F31)	6.0 (936)		7.7 (753)		<0.001^b^	*V* = 0.03^Δ^
Mood [affective] disorders (manic and bipolar affective disorders excluded) (F32–F39)	24.0 (3,764)		11.0 (1,075)		<0.001^b^	*V* = 0.16^Δ^
Neurotic, stress-related, and somatoform disorders (F40–F48)	13.0 (2,029)		7.8 (763)		<0.001^b^	*V* = 0.08^Δ^
Disorders of adult personality and behavior (F60–F69)	10.4 (1,632)		7.3 (714)		<0.001^b^	*V* = 0.05^Δ^
Other mental disorders	3.2 (497)		3.9 (384)		0.001^b^	*V* = 0.02^Δ^
Other non-mental disorders	1.4 (223)		2.7 (268)		<0.001^b^	*V* = 0.05^Δ^
**Comorbidity F10–F19, % (n)**	11.2 (1,758)		10.8 (1,056)		0.292^b^	–
**Comorbidity F60–F69, % (n)**	15.3 (2,394)		10.6 (1,033)		<0.001^b^	*V* = 0.07^Δ^
**HoNOS scores at admission, Mean (SD) Mdn (IQR)**						
Overactive, aggressive, disruptive, or agitated behavior	0.8 (1.2)	0 (2)	1.7 (1.5)	2 (3)	<0.001^a^	*r* = −0.32^ΔΔΔ^
Non-accidental self-injury	0.7 (1.3)	0 (1)	0.6 (1.2)	0 (0)	<0.001^a^	*r* = −0.04^Δ^
Problem drinking or drug-taking	1.3 (1.6)	0 (3)	1.2 (1.6)	0 (3)	<0.001^a^	*r* = −0.04^Δ^
Cognitive problems	0.7 (1.1)	0 (1)	1.4 (1.5)	1 (3)	<0.001^a^	*r* = −0.22^ΔΔ^
Physical illness or disability problems	0.7 (1.2)	0 (1)	0.9 (1.4)	0 (2)	<0.001^a^	*r* = −0.08^Δ^
Problems associated with hallucinations and delusions	0.8 (1.3)	0 (1)	1.5 (1.6)	1 (3)	<0.001^a^	*r* = −0.24^ΔΔ^
Problems with depressed mood	2.3 (1.3)	3 (1)	1.8 (1.4)	2 (3)	<0.001^a^	*r* = −0.18^Δ^
Other mental and behavioral problems	2.2 (1.4)	2 (2)	2.1 (1.5)	2 (3)	0.003^a^	*r* = −0.02^Δ^
Problems with relationships	1.6 (1.3)	2 (3)	1.9 (1.4)	2 (2)	<0.001^a^	*r* = −0.12^Δ^
Problems with activities of daily living	1.1 (1.3)	1 (2)	1.6 (1.4)	2 (3)	<0.001^a^	*r* = −0.16^Δ^
Problems with living conditions	1.2 (1.4)	1 (2)	1.4 (1.5)	1 (3)	<0.001^a^	*r* = −0.06^Δ^
Problems with occupation and activities	1.7 (1.4)	2 (3)	1.8 (1.4)	2 (3)	<0.001^a^	*r* = −0.06^Δ^
Problems with psychotropic medication compliance (additional item)	0.9 (1.3)	0 (2)	1.6 (1.6)	1 (3)	<0.001^a^	*r* = −0.23^ΔΔ^
**Referral from, % (*n*)**						
Patient	20.5 (3,122)		2.3 (220)		<0.001^b^	*V* = 0.26^ΔΔ^
Family/relatives	3.8 (574)		2.5 (242)		<0.001^b^	*V* = 0.03^Δ^
General practitioner	10.1 (1,531)		22.0 (2,098)		<0.001^b^	*V* = 0.16^Δ^
General hospital	14.6 (2,217)		23.0 (2,190)		<0.001^b^	*V* = 0.11^Δ^
Outpatient psychiatrist	41.6 (6,330)		33.3 (3,178)		<0.001^b^	*V* = 0.08^Δ^
Psychiatric hospital	5.2 (797)		7.6 (729)		<0.001^b^	*V* = 0.05^Δ^
Civil justice/other justice authority	1.2 (179)		6.2 (594)		<0.001^b^	*V* = 0.14^Δ^
Other	3.1 (476)		3.0 (287)		0.603^b^	*V* < 0.01^Δ^
**Hospital, % (*n*)**						
Hospital 1	18.6 (2,938)		23.0 (2,247)		<0.001^b^	*V* = 0.09^Δ^
Hospital 2	25.0 (3,947)		19.6 (1,921)			
Hospital 3	36.5 (5,771)		41.5 (4,060)			
Hospital 4	19.9 (3,141)		15.9 (1,559)			
**Time of admission, % (*n*)**						
Regular service hours	64.2 (10,147)		57.3 (5,604)		<0.001^b^	*V* = 0.07^Δ^
Outside regular service hours	35.8 (5,650)		42.7 (4,183)			

Note: Effect size: ^Δ^small effect; ^ΔΔ^small to medium effect; and ^ΔΔΔ^medium effect.

HoNOS, Health of the Nations Outcome Scale; VH, voluntary hospitalisation; IH, involuntary hospitalization; IQR, interquartile range; Mdn, median; SD, standard deviation.

a Mann–Whitney *U* test.

b Pearson’s Chi-square.

The three models were first fitted on the 70% of observations chosen randomly from the completely observed sample (training subsample; *n* = 10,464), and their predictive power was tested on the remaining 30% (test subsample; *n* = 4,484). For more details on the characteristics of training and test subsamples, please see the accompanying Supplementary Material. Default hyperparameters were used (maximum depth of the model = 30, minimum number of observations per node = 20, complexity parameter (cp) = 0.01, and number of models for RFs = 100). At each node, the split was performed on the variable with the lowest Gini index. Beside predictive accuracy, balanced accuracy and validity area under curve (AUC), other performance statistics were calculated: sensitivity, specificity, positive predictive value (PPV), and negative predictive value (NPV).

Due to the large proportion of missing information, which varied between 0 and 23% depending on the variable, and in order to verify the robustness of our results, we performed sensitivity analyses. Missing data were imputed through multiple imputation using chain equations [56]. CART and RFs were retested on the imputed data (*N* = 25,584).

All statistical analyses were performed using IBM SPSS 26 and R 4.0.1 (mice, stat, rpart, and randomForest libraries).

## Results

Between January 1, 2013 and December 31, 2017, 25,790 hospitalizations were registered within the four hospitals. Two hundred and six cases were excluded from the analyses since the legal status of the hospitalization was unknown. Of the included 25,584 hospitalizations, 61.7% were voluntary and 38.3% involuntary. The total sample was composed of 51% women, with an average age of 45 years, single (50%), of Swiss nationality (66%) and with a primary diagnosis of schizophrenic (F20–F29; 24%) or depressive disorders (F32–F39; 19%).

### Socio-demographic, clinical, and referral process characteristics: subgroups comparisons

The associations between legal status of the hospitalization and the socio-demographic, clinical and referral process characteristics are presented in Table 1. Globally, the two subgroups differed significantly on all the characteristics with the exclusion of sex, nationality, and secondary diagnosis of addiction. However, only five variables reached an effect size of 0.2 (small to medium effect) and only one exceeded the threshold of 0.3 (medium effect). Indeed, the IH group showed a considerably higher percentage of people affected by organic mental disorders (F00–F09) (*χ*^2^(1) = 1259.9; *p* < 0.001). Moreover, involuntary patients scored higher on four HoNOS items: overactive, aggressive, disruptive, or agitated behavior (*U* = 38316000.5; *z* = −48.5; *p* < 0.001), cognitive problems (*U* = 40715055.0; *z* = −31.5; *p* < 0.001), problems associated with hallucinations and delusions (*U* = 41784750.5; *z* = −35.8; *p* < 0.001), and the problems with psychotropic medication compliance additional item (*U* = 34761181.5; *z* = −32.4; *p* < 0.001). Finally, a remarkably higher percentage of voluntary patients auto-referred themselves to the hospital (*χ*^2^(1) = 1663.5; *p* < 0.001).

### Predicting factors of involuntary hospitalization

The original data contained a large number of missing values, and only 58% (14,948 out of 25,584) of the observations were complete.

Table 2 shows the predictive statistics produced for the three models on the completely observed test subsample (*n* = 4,484). The three methods achieved very similar results, with RFs and logistic regression showing slightly better performances compared to CART. Predictive balanced accuracy ranged from 68% for CART to 72% for logistic regression. The three models showed better specificity then sensitivity. Indeed, on the test subsample they were able to correctly classify between 81 and 86% of actual cases of voluntary hospitalization and between 54 and 58% of actual cases of involuntary hospitalization.

**Table 2.** Comparison of models’ validity.

	CART	Random Forests	Logistic regression
Balanced accuracy (%)	68.0	71.0	72.0
Accuracy (%)	71.3	75.6	75.6
AUC	0.68	0.71	0.72
Sensitivity	0.54	0.57	0.58
Specificity	0.81	0.86	0.86
PPV	0.61	0.69	0.69
NPV	0.76	0.78	0.78

Note: AUC, Area Under Curve; PPV, Positive Predictive Value; NPV, Negative Predictive Value.

**Figure 1.**
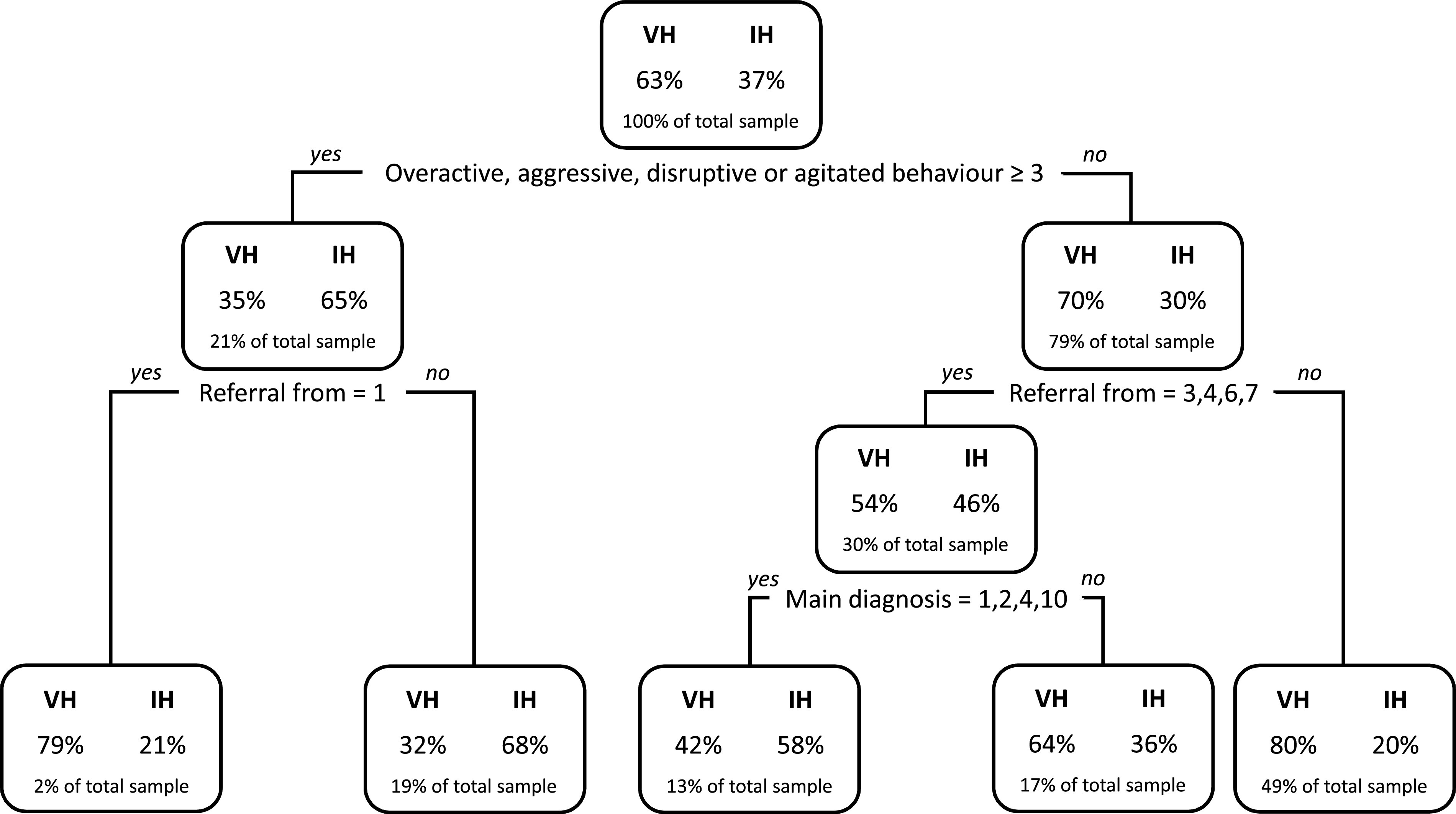
Classification and Regression Tree to predict involuntary hospitalizations on 70% of observations chosen randomly from completely observed sample (*N* = 10,464). Note: VH, voluntary hospitalization; IH, involuntary hospitalization. HoNOS scores: 0 = no problem; 1 = minor problem requiring no action; 2 = mild problem but definitely present; 3 = moderately severe problem; 4 = severe to very severe problem. Referral from: 1 = Patient; 2 = Family/relatives; 3 = General practitioner; 4 = General hospital; 5 = Outpatient psychiatrist; 6 = Psychiatric hospital; 7 = Civil justice/other justice authority; 8 = Other. Primary diagnosis: 1 = F00–F09; 2 = F10; 3 = F11–F19; 4 = F20–F29; 5 = F30–F31; 6 = F32–F39; 7 = F40–F48; 8 = F60–F69; 9 = Other mental disorders; 10 = Other non-mental disorders.

When we fitted a CART on the training subsample (*n* = 10,464), the resulting tree showed that only three parameters were essential to estimate the probability of being involuntarily admitted to hospital: the HoNOS score on the “Overactive, aggressive, disruptive or agitated behavior” item, who referred the patient to hospital and primary diagnosis (Figure 1).

The likelihood of coercion was higher for people with moderate to very severe aggressive behaviors (65%), especially if not auto-referred to hospital (68%). This percentage dropped to 21% when they referred themselves.

Among people showing none up to mild aggressiveness, there was a higher risk of involuntary admission if they were referred to hospital by a general practitioner, a general hospital, a psychiatric hospital or the civil justice (46%). A further increase was recorded if patients were affected by organic disorders (F00–F09), disorders due to alcohol use (F10), schizophrenia (F20–F29), and other non-mental disorders (58%). On the contrary, the odds of being involuntarily hospitalized decreased when the HoNOS score for aggressive behavior was lower than 3 (30%), and the patients referred themselves, or were referred by a family member/relative, an outpatient psychiatrist or others (20%).

**Figure 2.**
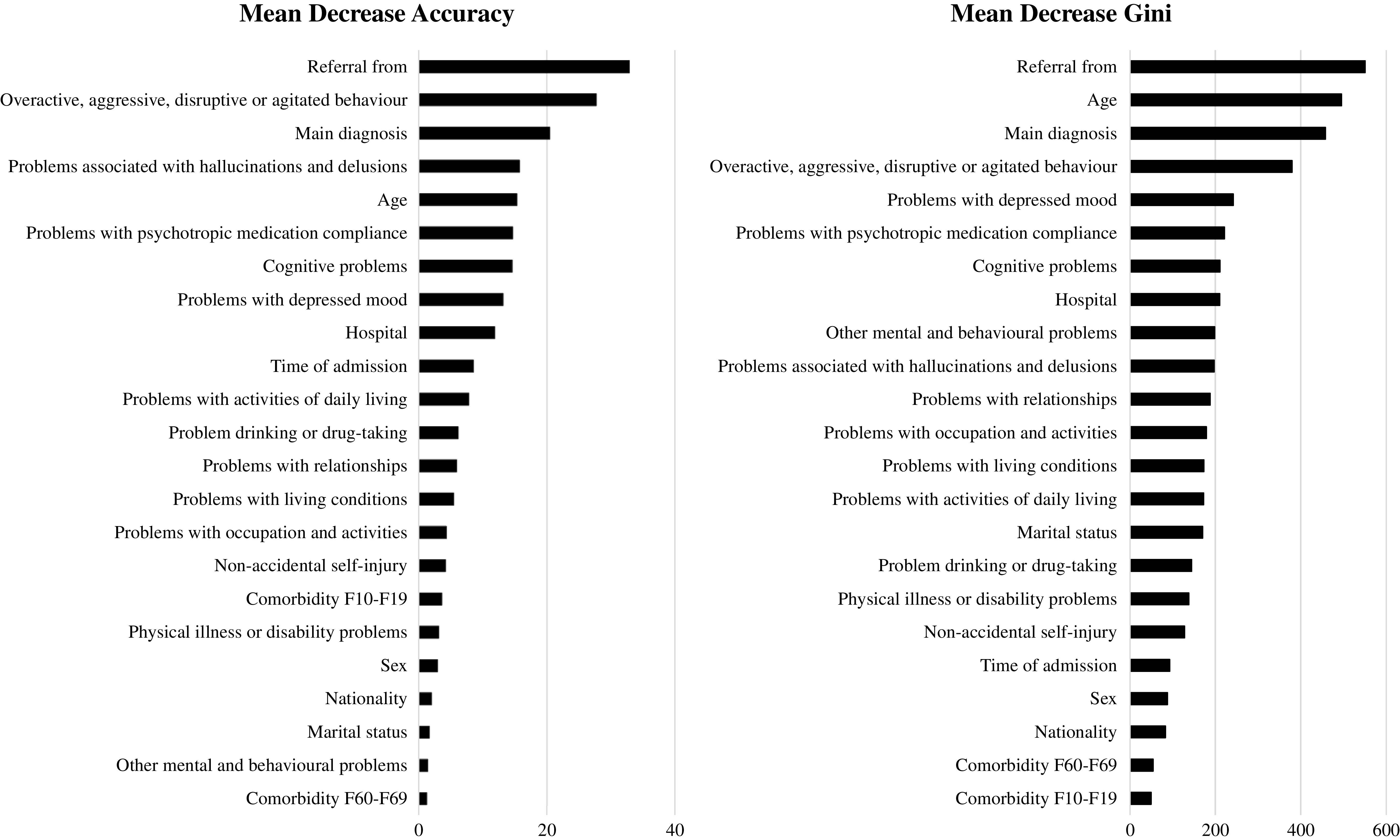
Mean decrease in accuracy and mean decrease in Gini coefficients (sorted decreasingly) in Random Forests model.

When RFs were fitted on the training subsample, the importance of each predictor was estimated based on its mean decrease in accuracy and its mean decrease in Gini coefficients (Figure 2). Based on these results, four factors emerged as the most important in both classifications: who referred the patient, the HoNOS score for “overactive, aggressive, disruptive, or agitated behavior,” primary diagnosis and age. The exclusion of one of these four variables would reduce either the accuracy of the model or its homogeneity.

Finally, of the 23 factors included in the logistic regression model, 15 were found to affect significantly the likelihood of being coerced to hospital (Table 3). However, in order to calculate the probability of an individual belonging to the IH group using this model, all the 23 characteristics must be available.

**Table 3.** Predictors of involuntary hospitalization: logistic regression analysis on 70% of observations chosen randomly from completely observed sample (*N* = 10,464).

Predicting factors	OR	95% CI		*p*
**Age**	1.01	1.00	1.01	0.012
**Sex** (ref. Male)				
Female	1.05	0.95	1.15	0.383
**Marital status** (ref. Single)				
Married/Registered partnership	0.92	0.80	1.07	0.274
Divorced/Separated	0.99	0.86	1.13	0.852
Widowed	1.12	0.87	1.44	0.394
**Nationality** (ref. Swiss)				
Other	1.28	1.15	1.42	<0.001
**Primary diagnosis at discharge** (ref. F00–F09)				
Mental and behavioral disorders due to use of alcohol (F10)	0.48	0.36	0.64	<0.001
Mental and behavioral disorders due to psychoactive substance use (F11–F19)	0.21	0.15	0.29	<0.001
Schizophrenia, schizotypal, and delusional disorders (F20–F29)	0.50	0.38	0.64	<0.001
Manic and bipolar affective disorders (F30–F31)	0.46	0.34	0.61	<0.001
Mood [affective] disorders (manic and bipolar affective disorders excluded) (F32–F39)	0.36	0.28	0.47	<0.001
Neurotic, stress-related and somatoform disorders (F40–F48)	0.44	0.33	0.57	<0.001
Disorders of adult personality and behavior (F60–F69)	0.38	0.29	0.51	<0.001
Other mental disorders	0.41	0.28	0.58	<0.001
Other non-mental disorders	0.62	0.43	0.91	0.013
**Comorbidity F10–F19**	0.80	0.68	0.94	0.008
**Comorbidity F60–F69**	1.13	0.98	1.31	0.088
**HoNOS**				
Overactive, aggressive, disruptive, or agitated behavior	1.33	1.28	1.39	<0.001
Non-accidental self-injury	1.07	1.02	1.11	0.002
Problem drinking or drug-taking	0.99	0.95	1.04	0.778
Cognitive problems	1.13	1.07	1.18	<0.001
Physical illness or disability problems	0.97	0.93	1.01	0.164
Problems associated with hallucinations and delusions	1.14	1.10	1.19	<0.001
Problems with depressed mood	0.80	0.76	0.83	<0.001
Other mental and behavioral problems	0.93	0.90	0.96	<0.001
Problems with relationships	1.04	0.99	1.08	0.102
Problems with activities of daily living	1.01	0.96	1.06	0.706
Problems with living conditions	1.06	1.02	1.10	0.005
Problems with occupation and activities	0.96	0.92	1.01	0.102
Problems with psychotropic medication compliance (additional item)	1.18	1.14	1.23	<0.001
**Referral from** (ref. Patient)				
Family/relatives	4.65	3.28	6.58	<0.001
General practitioner	13.48	10.60	17.29	<0.001
General hospital	11.12	8.77	14.22	<0.001
Outpatient psychiatrist	5.70	4.56	7.19	<0.001
Psychiatric hospital	8.86	6.73	11.73	<0.001
Civil justice/other justice authority	32.03	22.23	46.73	<0.001
Other	5.55	3.99	7.72	<0.001
**Hospital** (ref. Hospital 1)				
Hospital 2	0.65	0.56	0.74	<0.001
Hospital 3	0.91	0.80	1.03	0.133
Hospital 4	0.76	0.65	0.89	0.001
**Time of admission** (ref. Regular service hours)				
Outside regular service hours	1.51	1.37	1.67	<0.001
**Intercept**	0.65	0.45	0.92	0.015

Note: OR, Odds Ratio; CI, Confidence Interval.

### Sensitivity analyses

Due to the large proportion of missing information and in order to test the robustness of our results, we fitted both CART and RFs on 100 copies of data completed via multiple imputation. For CART the predictive accuracy on all scenarios ranged from 70 to 72%. The same CART shown in Figure 1 was obtained in 96 out of 100 replications of data completed via multiple imputation. For RFs the predictive accuracy for the 100 multiple imputation data versions varied between 73 and 75%.

## Discussion

The main aim of this study was to identify predicting factors of involuntary hospitalization using ML models. For this purpose, we analyzed our data using CART and RFs, and compared their predictive power with traditional logistic regression.

Our results showed that the performances of the three methods were very similar. CART showed the lowest predictive balanced accuracy (68%) but the most parsimonious model, allowing to estimate the probability of being involuntarily admitted to hospital with only three checks (aggressive behaviors, who referred the patient to hospital and primary diagnosis). Thanks to his binary structure, CART provided a prompt view of the most relevant predictors of involuntary admission and of their non-linear interactions. RFs and logistic regression achieved slightly higher results (71 and 72%), but at the cost of an increased complexity and a depleted practical feasibility of their models. In RFs, the mean decrease in accuracy and the mean decrease in Gini coefficients provided information about the importance of each variable and its impact on the accuracy and homogeneity of the model. However, nothing can be disclosed on how these factors interacted and affected each other. Logistic regression also made statistical interplay between risk factors difficult to model and interpret [13,39,40]. Moreover, more than 20 parameters had to be taken into account in order to estimate the probability of an individual to belong to the IH group.

The three models showed a greater ability to correctly classify real cases of voluntary hospitalization than to detect real cases of involuntary hospitalization. This result calls into question their actual use in real life and what margin of error should be considered ethically acceptable when dealing with a topic as sensitive as the use of coercion. Further research is mandatory to improve the sensitivity of the models.

The CART identified aggressive behaviors as the strongest predictor of coercion. People with moderate to very severe level of aggressiveness, especially if not auto-referred, showed the highest risk of involuntary hospitalization. Interestingly, the few aggressive patients who referred themselves had a low probability of compulsory admission. This suggests that, beside aggressiveness and agitation, uncooperativeness played a key role during the hospitalization process. RFs and logistic regression also identified aggression as one of the most important factors. Considering that in Switzerland dangerousness is not a prerequisite for involuntary admission but is only required to keep in hospital a voluntarily admitted person who wants to leave the institution, this finding cannot be considered as a mere consequence of the legal requirements in force. Several previous studies have already highlighted the key role of aggressive behaviors as a risk factor of coercion [15,17,18,20,21,23,32,41,44,57]. Aggressiveness may also affect the likelihood of being involuntarily admitted because it often requires the involvement of the police, which has been found to be another important risk factor of involuntary admission [17,20,44]. Perceived risk of danger to self or others has been found to be crucial in professionals’ decisions on involuntary admissions [58,59]. However, an accurate prediction of serious violence is very complex [60–62]. The use of a structured risk assessment during the admission process and the first days of treatment should be fostered [63–65]. Furthermore, the implementation of interventions and tools able to improve patients’ empowerment and participation in decision-making during psychiatric crisis, such as Joint Crisis Plan [66], should also be promoted in order to enhance cooperation, reduce the risk of aggressive behaviors and consequently reduce the use of coercion. Finally, dealing with aggression can be extremely challenging and stressful for staff [67]. A large number of referring physicians felt threatened by their patients when facing psychiatric emergency situations [68]. Thus, specific training programs on communication skills, management of aggressiveness and de-escalation techniques should be provided to all professionals involved in the referral process [63,64,69–71].

Indeed, referral process was identified as the second most important predictive factor in our decision tree. For people with lower levels of aggression, there was an increased risk of compulsion if they were referred to hospital by a general practitioner, a general hospital, a psychiatric hospital, or the civil justice. On the contrary, non-aggressive patients referred by themselves, by their relatives or by a psychiatrist were less likely to be involuntarily admitted regardless of diagnosis. Referral was also recognized as a strong predictor of coercion in both RFs and logistic regression. A previous study had already highlighted the association between physicians’ qualifications and coercion rates, showing that limiting the right to require involuntary admission to psychiatrists could reduce the use of compulsion [16]. Another study in Zurich, Switzerland, showed that referring general practitioners found it more difficult to apply legal criteria for involuntary admission and to assess its necessity [72]. GPs also provided the lowest quality commitment certificates [73,74]. In Cologne, Germany, Schmitz-Buhl et al. [13] found that the risk of involuntary treatment of patients with suicidal and self-harm behavior was higher in the two psychiatric hospitals that also showed a higher proportion of referrals from general emergency units. This result should call into question the organization of the on-call system in the canton of Vaud, which, despite the local high provision of psychiatrists (0.73/1,000 inhabitants), has been so far carried on solely by general practitioners. It is also worth to mention that psychiatric emergency services are not fully developed in the canton. More efforts should be made to enhance the psychiatric training of primary care physicians, who are often called outside regular service hours and in general emergency services to provide support and assess patients with mental health crisis.

The paramount need to develop trainings that improve the clinical knowhow and the aggressiveness management skills of all professionals involved in the admission process was also one of the recommendations that emerged from the EUNOMIA study for good clinical practice on involuntary hospitalization [75].

The last predictive factor identified by the CART was primary diagnosis. Among people with lower levels of aggressive behaviors and referred to hospital by a general practitioner, a general hospital, a psychiatric hospital or the civil justice, a higher risk for involuntary admission was registered if they were affected by organic disorders (F00–F09), disorders due to alcohol use (F10), schizophrenia (F20–F29), and other non-mental disorders. The logistic regression model upheld the strong predictive power of organic disorders, showing that they significantly increased the risk of being involuntarily admitted compared to all other diagnoses. New alternative treatments should be developed to better meet the special needs of this particular population. Indeed, several studies have already confirmed the relevance of this diagnosis as well as of the diagnosis of schizophrenia in predicting the risk of coercion [3,13–15,19,23,25,26,28,30,33,43,76]. Less conclusive are instead the findings concerning disorders due to alcohol use. Some earlier studies had found that being affected by substance abuse disorders, included alcohol, increased the odds of involuntary admission [3,28] while others showed a diminished risk for this population [13]. Finally, the increased risk associated with other non-mental disorders was not confirmed in the existing literature, according to which this diagnosis would have no impact on the likelihood of being involuntarily admitted [15]. The heterogeneity of the diagnostic categories included in this node makes it difficult to draw conclusions on which preventive measures should be fostered for this subgroup of patients. More homogeneous and therefore more informative subgroups could be obtained by including other parameters in the model.

Even though our main findings are mainly in line with previous studies, we cannot exclude that their representativeness might be affected by specific area-related characteristics, such as the local rate of involuntary admission, the specific regulation in place and, the local mental health organization and availability. According to the Swiss Health Observatory data, in 2016 the canton of Vaud was the canton with the highest rate of involuntary hospitalization in Switzerland, followed by Zurich and Geneva [48]. With 2.3 involuntary admission per 1,000 inhabitants, the canton of Vaud also ranked above most European countries, along with Germany and Austria [6]. Moreover, contrary to the majority of other Swiss cantons, in the canton of Vaud only some specific categories of medical doctors (general practitioners, psychiatrists, pediatricians, and on-call doctors) are allowed to order an involuntary inpatient treatment. Furthermore, as mentioned above, lack of capacity to consent to treatment and imminent danger, which are required in most countries, are not essential in Switzerland in order to admit someone involuntarily. Finally, model results could also be affected by the important variations existing among cantons and countries in term of psychiatric beds provision and community service development. Therefore, further national and international studies are needed to confirm the generalizability of our model.

### Strengths and limitations

The main strength of this study is the large sample size. Thanks to the available routinely collected data, we were able to include in the study all psychiatric admissions occurred between 2013 and 2017 in the canton of Vaud, Switzerland. The second main strength lies in the innovative methodologies deployed to analyze a large amount of data, without a priori selection of variables. The use of CART allowed us to overcome some of the main limitations of traditional statistical methods [13,39,40], providing a parsimonious and feasible hierarchical model with comparable predictive power.

Two main limitations should be reported. First, because of the retrospective nature of the study, only available routinely collected data could be included in our predictive models. Several important factors could not be taken into account. The introduction of these parameters into the models could have further improved their performances, especially their sensitivity. The second shortcoming was the large number of missing values in the original data. To overcome this limitation, sensitivity analyses were performed on 100 copies of data completed via multiple imputation. The results of both CART and RFs on the imputed data were almost identical to those obtained on the original data, confirming the robustness of our models.

## Conclusion

This study confirmed the essential role of aggressiveness, referral, and diagnosis as predictors of coercion. Identifying risk factors of involuntary admission is essential in order to efficiently target the development of professional training, preventive strategies, and alternative interventions. ML methodologies offer new effective tools to achieve this goal, providing accurate yet simple models that could be used in clinical practice.

## Data Availability

The data that support the findings of this study are available from the corresponding author upon reasonable request.

## Supplementary material

To view supplementary material for this article, please visit https://doi.org/10.1192/j.eurpsy.2021.2220 click here to view supplementary material
