# Supplementary material for: Predicting involuntary hospitalization in psychiatry: A machine learning investigation
Source: Eur Psychiatry. 2021 Jul 8;64(1):e48. doi: 10.1192/j.eurpsy.2021.2220 (PMC8316455; doi:10.1192/j.eurpsy.2021.2220)
Supplement: Supplementary file 1 [file S0924933821022203sup001.docx]

| Supplementary MaterialSocio-demographic, clinical and referral process characteristics: training and test completely observed subsamples (N=14948) | | | | | |  |
| --- | --- | --- | --- | --- | --- | --- |
| **Characteristics** | **Training**  **(n=10464; 70%)** | | **Test**  **(n=4484, 30%)** | | **p-value** |  |
| **Legal status,** Involuntary, % (n) | 36.8 (3850) | | 36.6 (1639) | | .780^a^ |  |
| **Age**, Mean (SD) Mdn (IQR) | 45.7 (18.1) | 43 (23.0) | 45.4 (17.9) | 43.0 (23.0) | .428^b^ |  |
| **Sex**, Male, % (n) | 47.9 (5016) | | 48.7 (2183) | | .401^a^ |  |
| **Marital status, % (n)** |  | |  | |  |  |
| Single | 48.6 (5081) | | 47.5 (2131) | | .091^a^ |  |
| Married/Registered partnership | 22.0 (2304) | | 22.2 (997) | |  |  |
| Divorced/Separated | 23.5 (2462) | | 25.0 (1123) | |  |  |
| Widowed | 5.9 (617) | | 5.2 (233) | |  |  |
| **Nationality,** Swiss, % (n) | 67.0 (7014) | | 67.2 (3013) | | .844^a^ |  |
| **Primary diagnosis at discharge (ICD-10), % (n)** |  | |  | |  |  |
| Organic, including symptomatic, mental disorders (F00-F09) | 7.1 (746) | | 6.8 (306) | | .504^a^ |  |
| Mental and behavioural disorders due to use of alcohol (F10) | 10.1 (1060) | | 10.1 (453) | | .959^a^ |  |
| Mental and behavioural disorders due to psychoactive substance use (F11-F19) | 8.2 (858) | | 7.9 (352) | | .473^a^ |  |
| Schizophrenia, schizotypal and delusional disorders (F20-F29) | 23.2 (2425) | | 23.7 (1064) | | .463^a^ |  |
| Manic and bipolar affective disorders (F30-F31) | 6.7 (705) | | 6.6 (294) | | .685^a^ |  |
| Mood [affective] disorders (manic and bipolar affective disorders excluded) (F32-F39) | 19.8 (2070) | | 19.2 (860) | | .395^a^ |  |
| Neurotic, stress-related and somatoform disorders (F40-F48) | 11.0 (1147) | | 10.8 (484) | | .763^a^ |  |
| Disorders of adult personality and behaviour (F60-F69) | 9.3 (977) | | 10.0 (450) | | .183^a^ |  |
| Other mental disorders | 2.6 (270) | | 2.8 (125) | | .469^a^ |  |
| Other non-mental disorders | 2.0 (206) | | 2.1 (96) | | .493^a^ |  |
| **Comorbidity F10-F19, % (n)** | 11.9 (1244) | | 12.0 (538) | | .849^a^ |  |
| **Comorbidity F60-F69, % (n)** | 15.1 (1579) | | 14.3 (643) | | .238^a^ |  |
| **HoNOS scores at admission, Mean (SD) Mdn (IQR)** |  | |  | |  |  |
| Overactive, aggressive, disruptive or agitated behaviour | 1.1 (1.4) | 0 (2) | 1.1 (1.4) | 0 (2) | .442^b^ |  |
| Non-accidental self-injury | 0.6 (1.2) | 0 (1) | 0.6 (1.2) | 0 (1) | .744^b^ |  |
| Problem drinking or drug-taking | 1.2 (1.6) | 0 (3) | 1.2 (1.6) | 0 (3) | .424^b^ |  |
| Cognitive problems | 0.9 (1.3) | 0 (2) | 0.9 (1.3) | 0 (2) | .144^b^ |  |
| Physical illness or disability problems | 0.8 (1.3) | 0 (2) | 0.8 (1.3) | 0 (2) | .726^b^ |  |
| Problems associated with hallucinations and delusions | 0.9 (1.4) | 0 (2) | 1.0 (1.4) | 0 (2) | .518^b^ |  |
| Problems with depressed mood | 2.2 (1.3) | 2 (2) | 2.2 (1.3) | 2 (2) | .605^b^ |  |
| Other mental and behavioural problems | 2.1 (1.4) | 2 (2) | 2.1 (1.4) | 2 (2) | .913^b^ |  |
| Problems with relationships | 1.6 (1.3) | 2 (3) | 1.6 (1.3) | 2 (3) | .781^b^ |  |
| Problems with activities of daily living | 1.2 (1.3) | 1 (2) | 1.3 (1.3) | 1 (2) | .731^b^ |  |
| Problems with living conditions | 1.2 (1.4) | 0 (2) | 1.2 (1.4) | 0 (2) | .926^b^ |  |
| Problems with occupation and activities | 1.6 (1.3) | 2 (3) | 1.6 (1.3) | 2 (3) | .330^b^ |  |
| Problems with psychotropic medication compliance (additional item) | 1.1 (1.4) | 0 (2) | 1.1 (1.4) | 0 (2) | .266^b^ |  |
| **Referral from, % (n)** |  | |  | |  |  |
| Patient | 13.6 (1418) | | 14.4 (644) | | .188^a^ |  |
| Family/relatives | 2.8 (290) | | 3.2 (145) | | .123^a^ |  |
| General practitioner | 15.2 (1590) | | 15.3 (687) | | .844^a^ |  |
| General hospital | 16.8 (1757) | | 16.5 (740) | | .666^a^ |  |
| Outpatient psychiatrist | 40.5 (4234) | | 39.1 (1753) | | .118^a^ |  |
| Psychiatric hospital | 5.6 (581) | | 6.1 (275) | | .162^a^ |  |
| Civil justice/ other justice authority | 2.7 (278) | | 2.3 (104) | | .231^a^ |  |
| Other | 3.0 (316) | | 3.0 (136) | | .966^a^ |  |
| **Hospital, % (n)** | |  | |  | |  |
| Hospital 1 | | 27.6 (2887) | | 27.1 (1213) | | .522^a^ |
| Hospital 2 | | 21.6 (2261) | | 21.9 (984) | |  |
| Hospital 3 | | 37.3 (3899) | | 36.7 (1644) | |  |
| Hospital 4 | | 13.5 (1417) | | 14.3 (643) | |  |
| **Time of admission, % (n)** | |  | |  | |  |
| Regular service hours | | 63.2 (6611) | | 63.1 (2829) | | .919^a^ |
| Outside regular service hours | | 36.8 (3853) | | 36.9 (1655) | |  |

Note. SD Standard Deviation, Mdn median, IQR interquartile range. ^a^ Pearson’s Chi-square. ^b^ Mann-Whitney U Test.
